# Nonlinear relationship between blood urea nitrogen to albumin ratio and in-hospital mortality in non-diabetic patients with non-ST-segment elevation myocardial infarction

**DOI:** 10.3389/fnut.2025.1499093

**Published:** 2025-04-28

**Authors:** Lixia Yuan, Wensen Yao

**Affiliations:** ^1^Department of Cardiac Rehabilitation, The Seventh People's Hospital of Zhengzhou, Zhengzhou, China; ^2^Department of Geriatrics and Special Medical Treatment, The First Hospital of Jilin University, Changchun, China

**Keywords:** blood urea nitrogen to albumin ratio, bar, NSTEMI, nonlinear, saturation effect, biomarker

## Abstract

**Background:**

The blood urea nitrogen (BUN) to albumin (ALB) ratio (BAR) is a novel biomarker that reflects both nutritional and inflammatory status and has been linked to the prognosis of various acute and chronic diseases. However, studies on its association with in-hospital prognosis in patients with non-ST-segment elevation myocardial infarction (NSTEMI) remain limited. Therefore, this study aimed to evaluate the relationship between BAR and in-hospital mortality in patients with NSTEMI.

**Methods:**

This study included 772 non-diabetic NSTEMI patients. The predictive performance was assessed using the area under the receiver operating characteristic (ROC) curve (AUC). Multivariable logistic regression was performed to identify the independent risk factors of in-hospital mortality. Subgroup analyses were conducted to evaluate the association between BAR and in-hospital mortality across different patient subgroups. Restricted cubic spline (RCS) function was applied to examine the nonlinear relationship between BAR and in-hospital mortality, and the two-piecewise logistic regression model was used for threshold effects analysis.

**Results:**

A total of 40 patients died during hospitalization. BAR exhibited strong predictive performance for in-hospital mortality (AUC = 0.83; 95% CI: 0.77–0.89). Multivariate analysis indicated that BAR was an independent risk factor for in-hospital mortality (OR = 1.06; 95% CI: 1.01–1.12), with a significant increase in mortality risk observed in most subgroups as BAR increased. A nonlinear relationship with a saturation effect was observed between BAR and in-hospital mortality (P for non-linearity = 0.002), with an inflection point of 8.51. Further two-piecewise logistic regression analysis revealed that when BAR was <8.51, the risk of in-hospital mortality increased significantly (OR = 1.69, 95% CI: 1.16–2.53), whereas when BAR was ≥8.51, the association was not statistically significant (OR = 0.99, 95% CI: 0.92–1.06).

**Conclusion:**

Baseline BAR serves as a simple, clinically useful prognostic biomarker of in-hospital mortality in non-diabetic NSTEMI patients. Additionally, we identified a nonlinear relationship with saturation effect between BAR and in-hospital mortality.

## 1 Introduction

Acute coronary syndromes (ACS), including ST-segment elevation myocardial infarction (STEMI), non-ST-segment elevation myocardial infarction (NSTEMI) and unstable angina, represent a major cause of mortality worldwide. Each year, over 7 million people worldwide are diagnosed with ACS, with over 1 million hospitalizations occurring in the United States alone (1). Compared with STEMI and unstable angina, NSTEMI incidence and hospitalization rates have risen rapidly in recent years. Although in-hospital mortality has declined, it remains concerning (2). In China, NSTEMI admission rates increased from 0.3 per 100,000 in 2001 to 3.3 per 100,000 in 2011 (3). Chinese study found that NSTEMI hospitalization rates nearly tripled from 2007 to 2012 (8.1 to 33.5%), while in-hospital mortality declined from 6.4 to 5.3% in men and from 11.6 to 8.7% in women (4). Studies from various countries and regions report NSTEMI in-hospital mortality rates ranging from 5.1–6.3% (5–7). Limited and unevenly distributed healthcare resources pose greater challenges for China and other low-and middle-income countries in reducing NSTEMI in-hospital mortality and improving prognosis compared with developed countries. Therefore, identifying early prognostic biomarkers and establishing risk stratification for NSTEMI are crucial for improving patient outcomes.

Blood urea nitrogen (BUN) is an essential marker of renal function and protein metabolism, whereas serum albumin (ALB) is widely employed to evaluate nutritional status and chronic inflammation. Both biomarkers are extensively used in clinical practice and are easily accessible. Increasing evidence suggests that elevated BUN levels and decreased ALB levels are significantly linked to poorer prognosis in patients with acute myocardial infarction (AMI) (8–11). Moreover, the BUN to ALB ratio (BAR), a novel biomarker integrating the characteristics of BUN and ALB, reflects the nutritional and inflammatory status of the body and has been demonstrated to be a reliable independent prognostic predictor in various diseases, including sepsis, chronic heart failure, and pulmonary embolism (12–14). The BAR may serve as a more effective comprehensive assessment tool than BUN or serum ALB alone for evaluating malnutrition, inflammatory status, and hepatic and renal reserve function in disease severity assessment (14).

Multiple studies have identified a correlation between BAR and both the diagnosis and prognosis of cardiovascular injury. A prospective cohort study involving 1,123 patients demonstrated that BAR is an independent risk factor for the development of left ventricular aneurysm (LVA) following percutaneous coronary intervention (PCI) in acute STEMI patients and has predictive value for diagnosing post-PCI LVA (15). A retrospective cohort study by Zhao et al. investigating the predictive value of BAR for long-term mortality in AMI patients revealed that those in the high BAR group had longer intensive care unit (ICU) stays and significantly higher 1-, 2-, 3-, and 4-year mortality rates than those in the low BAR group (*p* < 0.001). Furthermore, BAR was identified as a readily accessible biomarker independently predicting long-term mortality in AMI patients (16). A prospective study by Sevdımbas et al. involving 415 NSTEMI patients at initial admission demonstrated that BAR is an independent risk factor for 30-day mortality in NSTEMI patients (17). However, the association between BAR and in-hospital mortality remains unexplored in Chinese NSTEMI patients. Furthermore, the linear and nonlinear associations between BAR and in-hospital mortality in non-diabetic NSTEMI patients have not been investigated. Therefore, this study aims to: (1) comprehensively assess the correlation between BAR and in-hospital mortality among Chinese patients with NSTEMI; and (2) investigate the linear or nonlinear relationship between BAR and in-hospital mortality.

## 2 Materials and methods

### 2.1 Study design and patients

This was a single-center retrospective study. The data for this study were retrieved from a publicly available dataset (accessible at https://peerj.com/articles/14346/), provided by Zhao et al. (18). The original study was approved by the Ethics Committee of Zhongda Hospital affiliated to Southeast University (2020ZDSYLL164-P01). All authors have waived their copyright to the original research data. Since our study was a retrospective analysis of reused data aimed at improving clinical prognosis, no additional ethical approval was required.

The original study enrolled 774 NSTEMI patients without diabetes, who were hospitalized in the Cardiology Department of Zhongda Hospital from March 2012 to December 2018. Inclusion criteria: (1) Age > 18 years old; (2) First-time diagnosis of NSTEMI. Exclusion criteria: (1) Pregnant or lactating women; (2) Severe conditions such as advanced malignancies or an expected survival of <3 months; (3) Diabetes mellitus.

**Figure 1:**
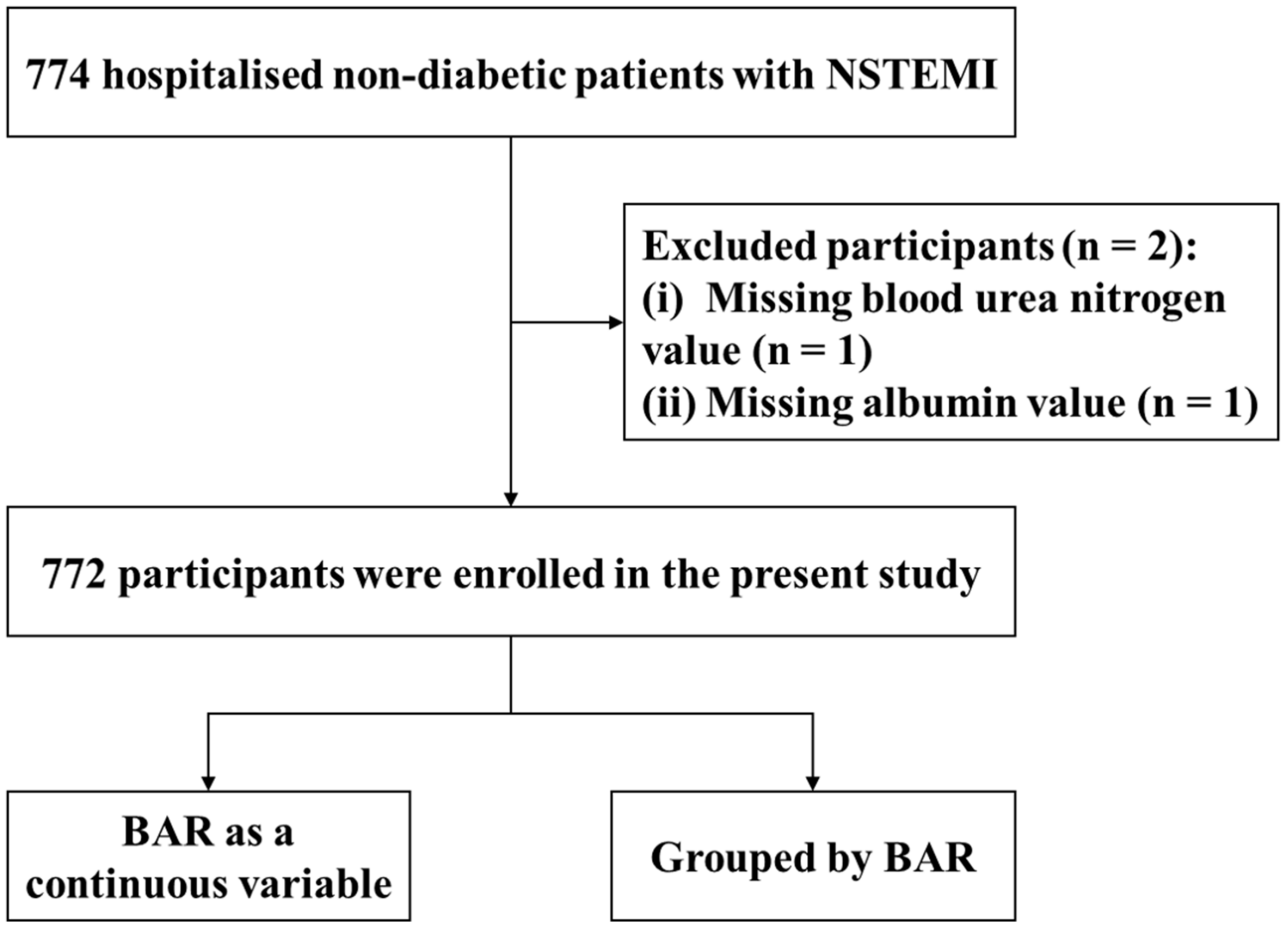
Research flow chart.

In the present study, we additionally excluded two participants due to missing BUN and ALB values (Figure 1). Ultimately, 772 non-diabetic NSTEMI patients were included in this study, of whom 732 survived to discharge and 40 died in hospital.

### 2.2 Data extraction

Patient data collected for this study, based on literature review and dataset availability, included demographics, vital signs, laboratory findings, and medications. These variables include age, sex, hypertension, chronic kidney disease (CKD), smoking, alcohol, systolic blood pressure (SBP), diastolic blood pressure (DBP), left ventricular ejected fraction (LVEF), white blood cells (WBC), neutrophils, lymphocytes, monocytes, platelet (PLT), hemoglobin (Hb), D-dimer, alanine aminotransferase (ALT), aspartate aminotransferase (AST), low-density lipoprotein cholesterol (LDL-C), high-density lipoprotein cholesterol (HDL-C), triglyceride (TG), total cholesterol (TC), uric acid (UA), creatinine, cardiac troponin I (cTnI), blood urea nitrogen (BUN), albumin (ALB), antihypertensive drug, lipid-lowering drug, aspirin, clopidogrel, nitrate, diuretics and β-blockers.

### 2.3 Definitions

The endpoint of this study was in-hospital mortality. BAR was calculated by dividing BUN (mg/L) at admission by the serum ALB (g/L). Hypertension was defined as a systolic blood pressure ≥ 140 mmHg or diastolic blood pressure ≥ 90 mmHg, measured on three separate occasions at rest without antihypertensive medication use (19). Chronic kidney disease (CKD) was defined as chronic renal structural and functional dysfunction (history of renal damage greater than 3 months) from a variety of causes, including pathological damage with normal and abnormal renal GFR, abnormal blood or urine composition, and abnormal imaging, or unexplained decline in GFR for more than 3 months (20).

### 2.4 Statistical analyses

Before analysis, data completeness was assessed. We performed multiple imputation (mice package) for variables with less than 30% missingness, generating a total of five sets of post-imputation data and selecting the optimal post-imputation dataset based on the Akaike Information Criterion (AIC) for the subsequent analyses. The distributions of all quantitative variables were analyzed using the Shapiro–Wilk test. According to the Shapiro–Wilk analysis, all quantitative data were non-normally distributed, presented as median (interquartile range), and compared using the Mann–Whitney test. Categorical variables were presented as counts and percentages, and differences between two groups were compared by the chi-squared test or the Fisher exact test.

ROC curve analyses were performed for BAR, BUN combined with ALB, BUN, and ALB to evaluate their predictive performance for in-hospital mortality, with the DeLong test used for AUC comparisons. The optimal cut-off value of BAR was determined using the maximum Youden index (sensitivity + specificity -1) and accordingly the patients were categorized into two subgroups (BAR <8.52 mg/g and BAR ≥8.52 mg/g). Least Absolute Shrinkage and Selection Operator (LASSO) regression was applied to select the potential predictors from 33 candidate variables. Variables screened by LASSO regression were then included in multivariate logistic regression analyses to assess the independent predictive role of BAR (as a continuous and categorical variable) for in-hospital death in non-diabetic NSTEMI patients. Subgroup analyses were used to explore whether the relationship between BAR and in-hospital mortality is consistent across subgroups of the population with different characteristics. Logistic regression model with restricted cubic spline (RCS) was used to evaluate the relationship between baseline BAR value (continuous variable) and the risk of in-hospital mortality. If non-linearity was detected, the “segmented” package was used to calculate the inflection point. Then a two-piecewise logistic regression model was fitted to calculate the threshold effect of the BAR on in-hospital mortality. All statistical analyses were conducted using R software (4.2.2). A *p*-value <0.05 (two-tailed) or a 95% confidence interval (CI) for the odds ratio excluding one was considered statistically significant.

## 3 Results

### 3.1 Baseline characteristics

The baseline characteristics of the study participants are summarized in Table 1. A total of 772 eligible patients were included in the study, with a median age of 76 years, of whom 31.2% were female. Among them, 732 (94.8%) survived to discharge, while 40 (5.2%) died during hospitalization.

**Table 1:** Baseline characteristics of patients with NSTEMI.

Variables	All patients (*n* = 772)	Survival group (*n* = 732)	Death group (*n* = 40)	*P*-value
Age (y)	76 [64, 85]	75 [64, 84]	87 [83, 91]	<0.001
Female, *n* (%)	241 (31.2)	223 (30.5)	18 (45.0)	0.079
Hypertension, *n* (%)	553 (71.6)	521 (71.2)	32 (80.0)	0.305
CKD, *n* (%)	65 (8.4)	57 (7.8)	8 (20.0)	0.016
Smoking, *n* (%)	289 (37.4)	280 (38.3)	9 (22.5)	0.066
Alcohol, *n* (%)	107 (13.9)	106 (14.5)	1 (2.5)	0.057
SBP (mmHg)	133 [120, 146]	134 [120, 147]	126 [106, 137]	0.003
DBP (mmHg)	80 [70, 86]	80 [70, 86]	75 [60, 82]	0.013
LVEF (%)	62 [52, 70]	63 [53, 70]	58 [46, 66]	0.025
**Laboratory tests**				
WBC (10^9^/L)	7.36 [6.07, 9.38]	7.31 [6.02, 9.27]	8.60 [6.60, 11.73]	0.010
Neutrophils (10^9^/L)	5.24 [3.99, 7.21]	5.22 [3.93, 7.08]	6.64 [4.80, 10.53]	0.002
Lymphocytes (10^9^/L)	1.34 [0.95, 1.77]	1.37 [0.98, 1.77]	0.82 [0.61, 1.46]	0.001
Monocytes (10^9^/L)	0.43 [0.32, 0.56]	0.43 [0.32, 0.56]	0.48 [0.33, 0.56]	0.415
PLT (10^9^/L)	187 [149, 227]	188 [150, 227]	184 [138, 235]	0.317
Hb (g/L)	132 [115, 146]	133 [117, 147]	112 [97, 125]	<0.001
D-dimer (mg/L)	0.18 [0.08, 0.47]	0.17 [0.08, 0.44]	0.62 [0.30, 3.05]	<0.001
ALT (U/L)	24.00 [16.00, 35.00]	24.00 [16.00, 35.00]	30.00 [13.00, 38.25]	0.219
AST (U/L)	33.00 [22.00, 63.00]	32.50 [22.00, 62.00]	47.50 [21.75, 128.75]	0.056
LDL-C (mmol/L)	2.64 [2.08, 3.17]	2.65 [2.12, 3.18]	2.24 [1.74, 2.90]	0.010
HDL-C (mmol/L)	1.05 [0.91, 1.22]	1.06 [0.92, 1.22]	0.92 [0.77, 1.17]	0.007
TG (mmol/L)	1.27 [0.91, 1.74]	1.27 [0.91, 1.74]	1.38 [0.95, 1.69]	0.396
TC (mmol/L)	4.31 [3.61, 5.01]	4.34 [3.64, 5.02]	3.83 [3.14, 4.68]	0.017
UA (μmol/L)	351.50 [282.00, 424.00]	350.00 [282.00, 421.00]	360.00 [263.75, 478.00]	0.449
Creatinine (μmol/L)	88.00 [70.00, 112.00]	87.00 [69.00, 109.00]	128.50 [104.50, 210.25]	<0.001
cTnI (ng/ml)	0.70 [0.13, 3.17]	0.67 [0.13, 2.95]	1.55 [0.16, 4.84]	0.255
BUN (mmol/L)	6.10 [4.70, 8.20]	5.95 [4.70, 7.90]	10.35 [8.52, 14.23]	<0.001
ALB (g/L)	37.25 [34.00, 40.20]	37.60 [34.30, 40.40]	33.90 [28.40, 37.00]	<0.001
BAR	4.61 [3.40, 6.47]	4.48 [3.37, 5.96]	9.06 [6.99, 13.75]	<0.001
**Medications**				
Antihypertensive drug, *n* (%)	538 (69.7)	522 (71.3)	16 (40.0)	<0.001
Lipid-lowering drug, *n* (%)	730 (94.6)	702 (95.9)	28 (70.0)	<0.001
Aspirin, *n* (%)	736 (95.3)	708 (96.7)	28 (70.0)	<0.001
Clopidogrel, *n* (%)	684 (88.6)	657 (89.8)	27 (67.5)	<0.001
Diuretics, *n* (%)	288 (37.3)	280 (38.3)	8 (20.0)	0.031
β-blockers, *n* (%)	623 (80.7)	603 (82.4)	20 (50.0)	<0.001
Nitrate, *n* (%)	611 (79.1)	589 (80.5)	22 (55.0)	<0.001

Data are presented as the *n* (%) or median (25th Percentile, 75th Percentile).

NSTEMI, non-ST-segment elevation myocardial infarction; CKD, chronic kidney disease; SBP, systolic blood pressure; DBP, diastolic blood pressure; LVEF, left ventricular ejected fraction; WBC, white blood cells; PLT, platelet; Hb, hemoglobin; ALT, alanine aminotransferase; AST, aspartate aminotransferase; LDL-C, low-density lipoprotein cholesterol; HDL-C, high-density lipoprotein cholesterol; TG, triglyceride; TC, total cholesterol; UA, uric acid; cTnI, cardiac troponin I; BUN, blood urea nitrogen; ALB, albumin; BAR, blood urea nitrogen to albumin ratio.

A comparison of the clinical characteristics between the two groups revealed that patients who died in hospital were older, had a higher prevalence of CKD, and had lower SBP, DBP, and LVEF than those who survived to discharge (*p* < 0.05). Regarding laboratory parameters, patients in the death group had higher levels of WBC count, neutrophil count, D-dimer, creatinine, BUN, and BAR, and lower levels of lymphocyte count, Hb, LDL-C, HDL-C, TC, and ALB (*p* < 0.05). Regarding medication use, patients in the death group had significantly lower usage rates of antihypertensive drugs, lipid-lowering drugs, aspirin, clopidogrel, diuretics, nitrate, and β-blockers compared to survivors (*p* < 0.05).

### 3.2 Comparing the predictive value of different predictors

**Figure 2:**
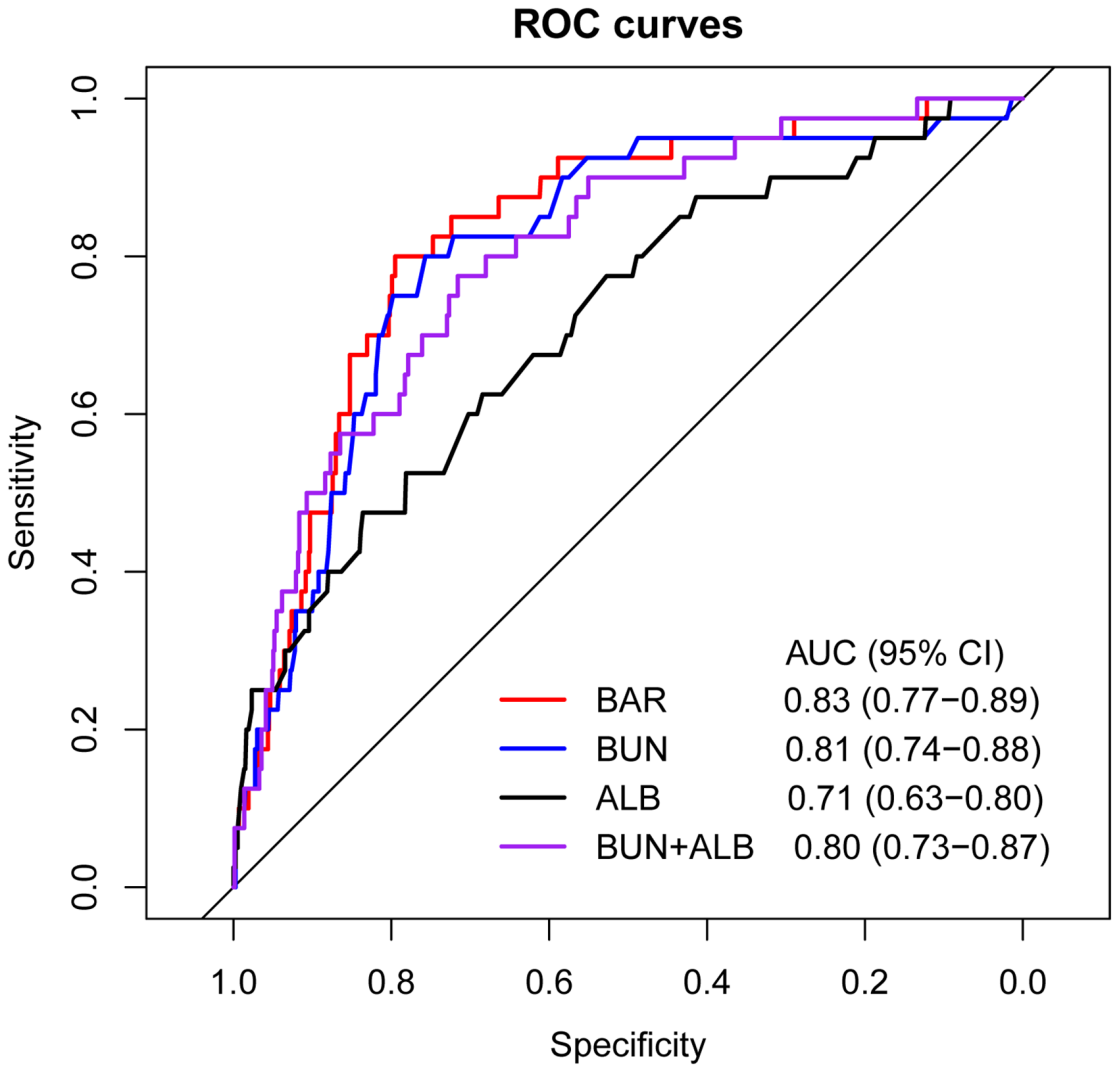
Receiver operating characteristic (ROC) curve by in-hospital mortality.

ROC curve analyses were conducted to evaluate and compare the predictive performance of BAR, BUN, ALB, and BUN combined with ALB for in-hospital mortality (Figure 2). As presented in Table 2, the AUC of BAR was 0.83 (95% CI: 0.77–0.89), with an optimal cut-off value of 6.65, corresponding to a sensitivity of 80.0% and a specificity of 79.5%. The AUC of BUN was 0.81 (95% CI: 0.74–0.88), with an optimal cut-off value of 7.95, yielding a sensitivity of 80.0% and a specificity of 75.7%. The AUC of ALB was 0.71 (95% CI: 0.63–0.80), with an optimal cut-off value of 32.55, yielding a sensitivity of 47.5% and a specificity of 83.6%. The AUC of BUN combined with ALB was 0.80 (95% CI: 0.73–0.87), with an optimal cut-off value of 0.045, yielding a sensitivity of 77.5% and a specificity of 71.6%. BAR exhibited superior predictive performance for in-hospital mortality compared with ALB (DeLong’s test *p* = 0.033). BAR, BUN, and BUN combined with ALB all exhibited good predictive performance for in-hospital mortality, with AUCs ≥0.8. The detailed results of the ROC analysis are given in Table 2.

**Table 2:** Analysis of the ROC curve for predictive power of in-hospital mortality.

	Cut-off	AUC (95% CI)	Sensitivity (%)	Specificity (%)	P for DeLong’s test
BUN (mmol/L)	7.95	0.81 (0.74–0.88)	80.0	75.7	
ALB (g/L)	32.55	0.71 (0.63–0.80)	47.5	83.6	
BUN+ALB	0.045	0.80 (0.73–0.87)	77.5	71.6	
BAR (mg/g)	6.65	0.83 (0.77–0.89)	80.0	79.5	
BAR vs. BUN					0.651
BAR vs. ALB					0.033
BAR vs. BUN+ALB					0.555
BUN+ALB vs. BUN					0.868
BUN+ALB vs. ALB					<0.001
BUN vs. ALB					0.087

AUC, area under the receiver operating characteristic curve; CI, confidence interval; BUN, blood urea nitrogen; ALB, albumin; BAR, blood urea nitrogen to albumin ratio.

### 3.3 Variable selection based on the LASSO regression

**Figure 3:**
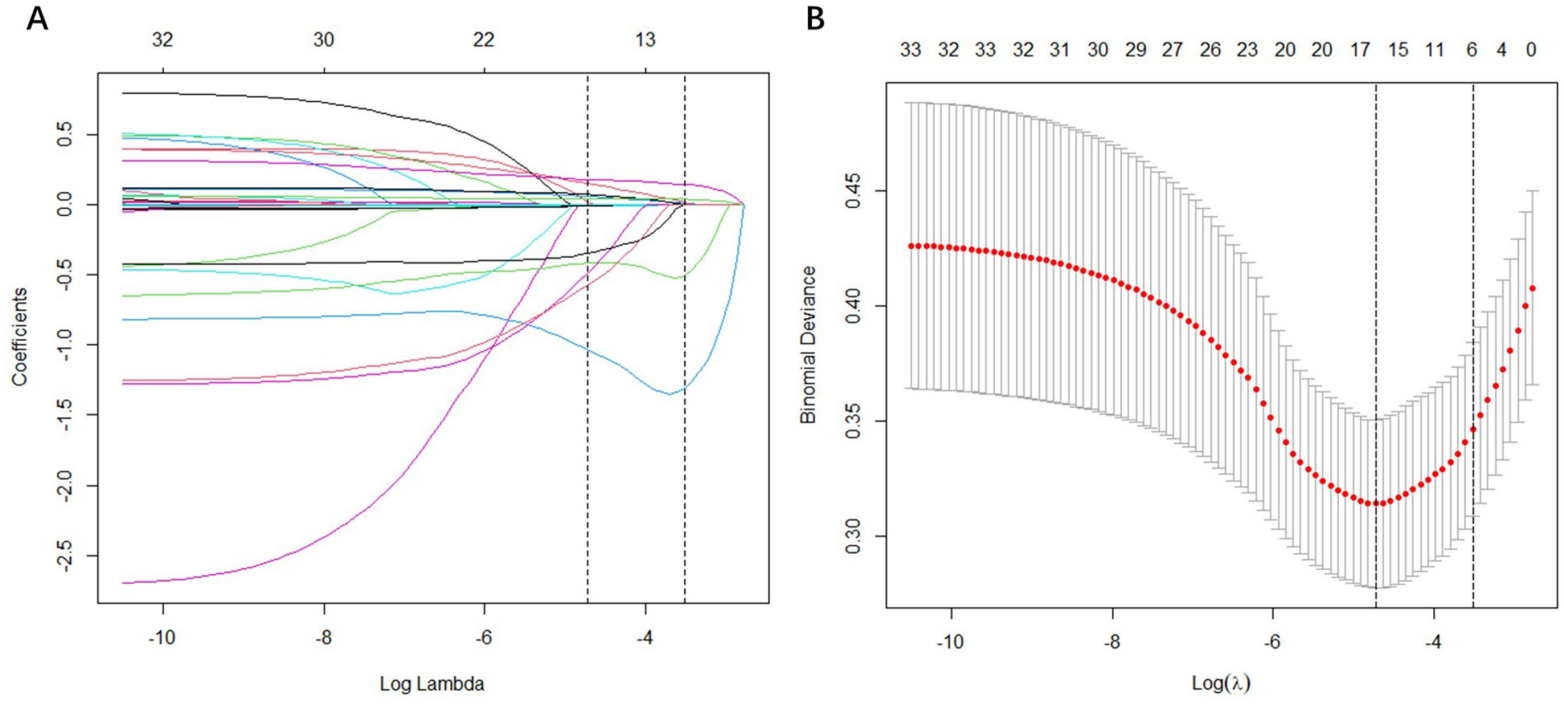
Feature selection using the least absolute shrinkage and selection operator (LASSO) binary logistic regression model. **(A)** LASSO coefficient profiles of the 33 baseline features; **(B)** The tuning parameter (λ) selection in the LASSO model using 10-fold cross-validation via minimum criteria. Vertical lines were drawn at the value selected using 10-fold cross-validation, where optimal λ resulted in 6 non-zero coefficients.

LASSO regression was applied to perform the initial selection of predictors. The LASSO regression incorporated 33 candidate variables (all variables except “BUN” and “ALB” in Table 1), and based on the LASSO regression analysis, six variables with non-zero coefficients were selected as predictors: age, TG, D-dimer, BAR, aspirin and lipid-lowering drug (Figure 3).

### 3.4 Risk factors associated with in-hospital mortality in nondiabetic patients with NSTEMI

As shown in Table 3, six variables screened by LASSO regression were included in the univariate logistic regression analysis and those variables that were statistically significant (*p* < 0.05) in the univariate analysis were subsequently included in the multivariate logistic regression model. Multivariate analysis identified age, TG, D-dimer, aspirin and BAR as independent risk factors for in-hospital mortality in non-diabetic patients with NSTEMI. In the logistic regression model finally adjusted for age, TG, D-dimer, aspirin, and lipid-lowering drug, BAR remained a significant predictor of in-hospital mortality (OR = 1.06, 95% CI: 1.01–1.12, *p* = 0.030).

**Table 3:** Univariate and multivariate logistic regression analyses.

Variables	Univariate analysis		Multivariate analysis	
	OR (95% CI)	*P*-value	OR (95% CI)	*P*-value
Age (y)	1.11 (1.07–1.16)	<0.001	1.11 (1.06–1.16)	<0.001
TG (mmol/L)	1.37 (1.16–1.63)	<0.001	1.39 (1.07–1.98)	0.046
D-dimer (mg/L)	1.62 (1.35–1.94)	<0.001	1.36 (1.03–1.76)	0.021
Aspirin	0.08 (0.04–0.17)	<0.001	0.17 (0.06–0.47)	<0.001
Lipid-lowering drug	0.10 (0.05–0.22)	<0.001	0.40 (0.15–1.16)	0.076
BAR^a^ (mg/g)	1.13 (1.08–1.18)	<0.001	1.06 (1.01–1.12)	0.030
BAR^b^ (mg/g)				
<6.65	Reference		Reference	
≥6.65	13.36 (6.48–30.38)	<0.001	5.90 (2.52–15.32)	<0.001

OR, odds ratio; CI, confidence interval; BUN, blood urea nitrogen; ALB, albumin; TG, triglyceride; BAR, blood urea nitrogen to albumin ratio.

a BAR as a continuous variable.

b BAR as a categorizing variable.

Based on the optimal cut-off value of BAR, NSTEMI patients were stratified into a high BAR group (BAR ≥ 6.65 mg/g) and a low BAR group (BAR < 6.65 mg/g). Multivariate analysis was then conducted to further investigate the association between BAR and in-hospital mortality (Table 3). The results showed that after adjusting for confounders, the risk of in-hospital mortality in patients in the high BAR group was 5.9 times higher than that in patients in the low BAR group (OR = 5.90, 95%CI: 2.52–15.32, *p* < 0.001).

### 3.5 Subgroup analyses

**Figure 4:**
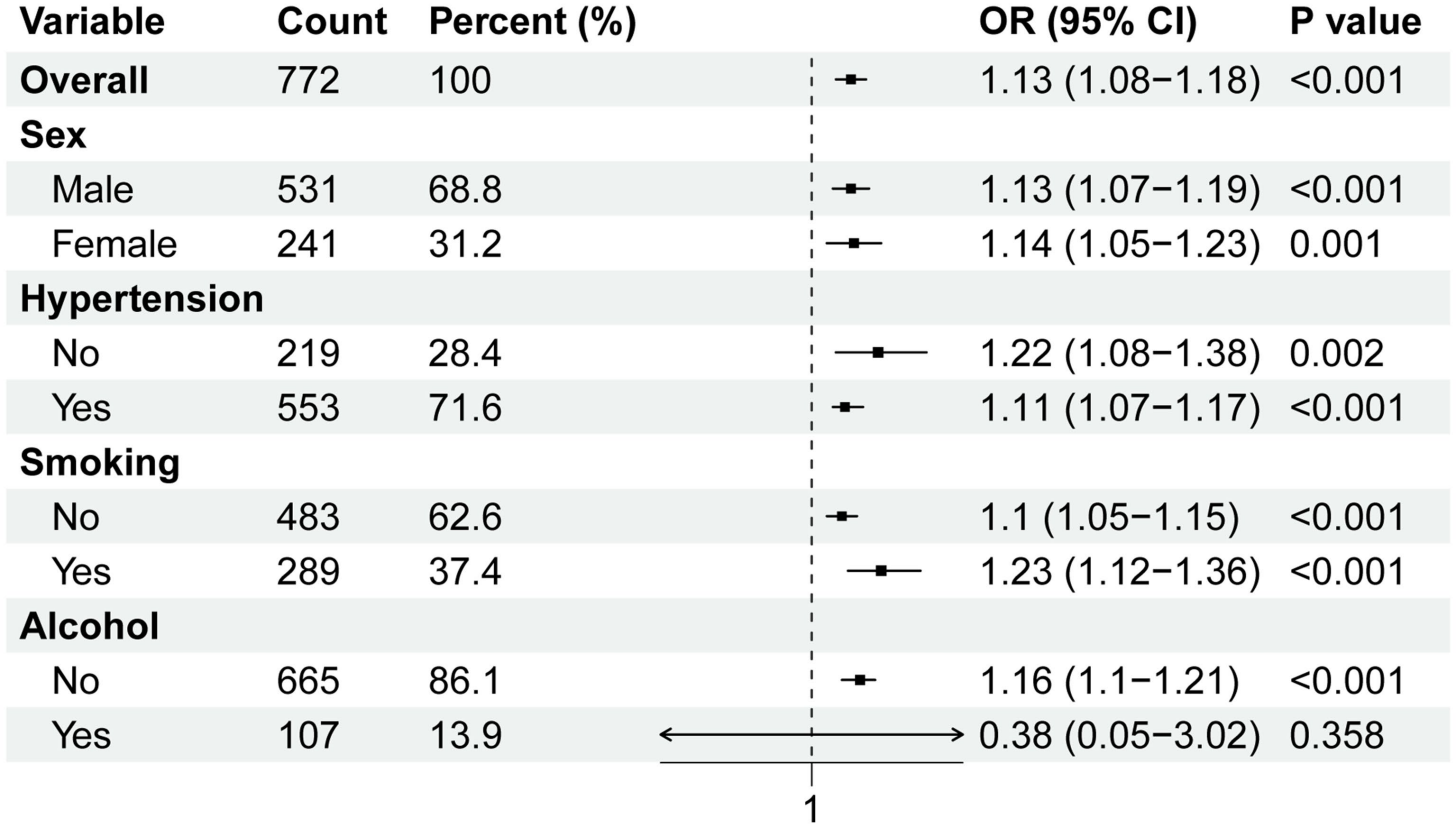
Subgroup analyses for the association of BAR with in-hospital mortality.

Subgroup analyses were conducted to assess the association between BAR and in-hospital mortality across different population subgroups. We performed subgroup analyses according to sex, hypertension, smoking, and alcohol consumption, which showed that the risk of in-hospital mortality increased significantly with increasing BAR in most subgroups (Figure 4). In the alcoholic drinking subgroup, BAR was not significantly associated with the risk of in-hospital mortality.

### 3.6 Exploring the nonlinear relationship between BAR and in-hospital mortality

**Figure 5:**
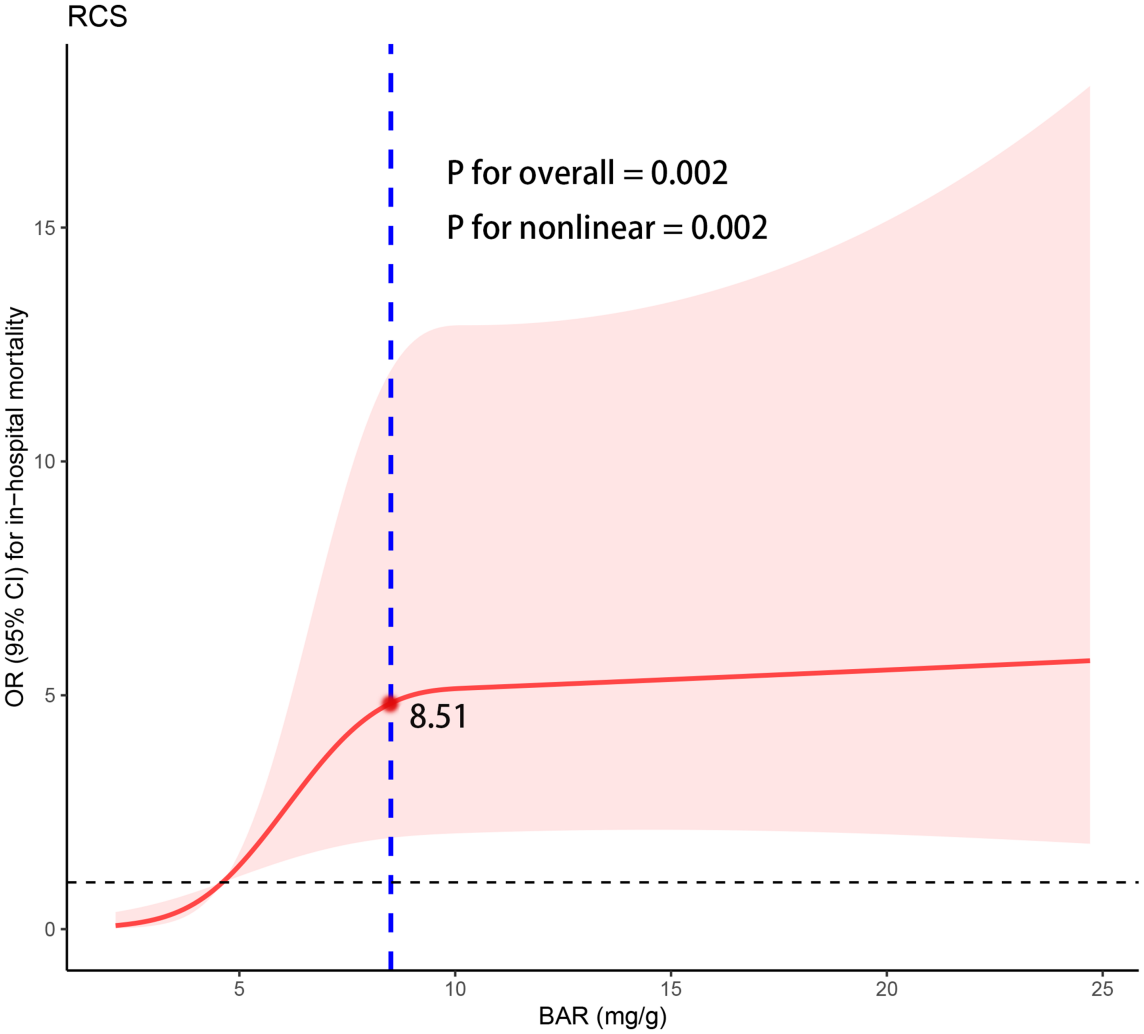
The RCS regression between the BAR with in-hospital mortality. OR, odds ratio; CI, confidence interval; RCS, restricted cubic spline; BAR, blood urea nitrogen to albumin ratio.

To further explore the nonlinear relationship between BAR and in-hospital mortality, multivariable-adjusted (adjusted for age, TG, D-dimer and aspirin) RCS model based on logistic regression was performed (Figure 5). A nonlinear relationship with a saturation effect between BAR and in-hospital mortality was observed (P for non-linearity = 0.002), with the inflection point calculated as 8.51 using the “segmented” package.

We then performed a two-piecewise logistic regression (Table 4), which showed that the risk of in-hospital mortality increased significantly with increasing BAR when the BAR value was <8.51 (OR = 1.69, 95% CI: 1.16–2.53), but the correlation was not statistically significant when the BAR value was ≥8.51 (OR = 0.99, 95% CI: 0.92–1.06) (Figure 5).

**Table 4:** Two-piecewise logistic regression.

BAR (mg/g)	Events/total	OR (95% CI)	*P*-value
<8.51	16/650	1.69 (1.16–2.53)	0.007
≥8.51	24/122	0.99 (0.92–1.06)	0.720

BAR, blood urea nitrogen to albumin ratio; OR, Odds Ratio; CI, confidence interval.

## 4 Discussion

The majority of cardiovascular-related deaths occur in low-and middle-income countries. Due to the rising global prevalence of NSTEMI and its unfavorable in-hospital and out-of-hospital outcomes, there is a pressing need for an efficient and widely applicable biomarker to stratify NSTEMI patients by risk and to develop personalized treatment strategies aimed at improving both short-and long-term prognosis. This study identified BAR as a clinically valuable predictor of in-hospital mortality in patients with NSTEMI. The key findings of this study are summarized as follows: (1) An elevated baseline BAR at admission independently predicted in-hospital mortality in non-diabetic NSTEMI patients; (2) BAR demonstrated strong predictive value for in-hospital mortality, and its monitoring is beneficial for the early identification of high-risk patients; (3) Notably, for the first time, we identified a nonlinear relationship between BAR and in-hospital mortality, further determining an inflection point (8.51 mg/g). Specifically, to the left of the inflection point, in-hospital mortality risk increased significantly with rising BAR; however, to the right, a saturation effect was observed.

BUN is a protein metabolite primarily produced in the liver and excreted by the kidney, serving as a crucial biomarker for assessing renal function and metabolic status. The primary causes of elevated BUN levels include: (1) Enhanced proteolytic metabolism and increased BUN synthesis; (2) Activation of the renin–angiotensin–aldosterone and sympathetic nervous systems due to hypoperfusion, subsequently increasing BUN reabsorption; and (3) Renal impairment. Elevated BUN levels have been reported to reflect the severity of the disease as well as poor prognosis (21, 22). A Japanese study of 2,995 AMI patients demonstrated that elevated BUN levels independently predicted in-hospital mortality (9). Other studies have highlighted the predictive value of elevated BUN for long-term mortality in AMI patients (8, 23). Consistent with these findings, our study demonstrated an association between elevated BUN levels and increased in-hospital mortality in non-diabetic NSTEMI patients. Furthermore, ROC curve analysis indicated that BUN was comparable to BAR in predicting in-hospital mortality, suggesting its potential as a valuable serum biomarker for risk assessment in NSTEMI patients. ALB is a liver-synthesized protein and the most abundant protein in plasma. Beyond its well-established role in nutrition and colloid osmotic pressure homeostasis, ALB possesses several physiological properties, including antioxidant function, immunomodulation, antiplatelet aggregation, and anti-inflammatory activity (24, 25). Increasing evidence suggests that low ALB levels indicate a state of heightened inflammation, which is closely linked to the development and prognosis of various cardiovascular diseases. A cross-sectional study involving 1,552 cases and 6,680 controls demonstrated that low serum ALB concentrations were strongly associated with the development of AMI (26). An Israeli prospective study demonstrated that a decrease in ALB on admission was significantly associated with long-term all-cause mortality in AMI patients, exhibiting a “dose–response” relationship (27). Our study builds upon previous findings, demonstrating that ALB has a reasonable predictive value for in-hospital mortality risk in non-diabetic NSTEMI patients (AUC = 0.71, 95%CI: 0.63–0.80). The increased risk of in-hospital mortality in NSTEMI due to decreased serum ALB levels may be attributed to the diminished anti-inflammatory, antioxidant, and antithrombotic properties of ALB.

BAR is recognized as a novel biomarker of inflammation and nutritional status, integrating the characteristics of BUN and ALB. In recent years, BAR has emerged as a prognostic biomarker for various diseases, including heart failure, chronic obstructive pulmonary disease and gastrointestinal bleeding (13, 21, 28). Previous studies have reported the effect of BAR levels on AMI prognosis (15, 29, 30), and the incidence of left ventricular aneurysm (LVA) after PCI (15). Furthermore, in a retrospective cohort study of patients with acute ischemic stroke (AIS), Li et al. evaluated the predictive value of BAR for the risk of in-hospital death in patients with AIS and showed that serum BAR was an important biomarker for identifying patients with AIS who are at high risk of death (31). Yet only one specifically examined the detrimental effect of elevated BAR on in-hospital mortality in NSTEMI (17). In this study, BAR levels were significantly higher in deceased patients compared to survivors. Multivariate analysis identified BAR as an independent predictor of in-hospital mortality, while ROC analysis confirmed its good predictive value. Additionally, using RCS and a two-piecewise logistic regression model, we identified a nonlinear relationship and a saturation effect between BAR and in-hospital mortality. Specifically, when BAR <8.51, each 1-unit increase corresponded to a 69% rise in in-hospital mortality risk (OR = 1.69, 95% CI: 1.16–2.53). However, when BAR ≥8.51, further increases in BAR did not significantly alter the risk of in-hospital mortality (OR = 0.99, 95% CI: 0.92–1.06). The precise causes and underlying pathophysiological mechanisms linking elevated BAR levels to poorer prognosis remain uncertain. Therefore, we can only hypothesize potential mechanisms by focusing on the key components of the BAR, namely BUN and ALB (29). As previously discussed, elevated BUN levels and reduced ALB levels often indicate metabolic dysregulation, nutritional deficiencies, and systemic inflammation, which may explain the association between high BAR and poor prognosis across various diseases. STEMI patients frequently experience inadequate protein intake due to appetite loss or impaired digestive function, which disrupts albumin synthesis (32). And, post-myocardial infarction metabolic disturbances accelerate protein catabolism, further depleting albumin levels (33). Additionally, acute post-infarction events elicit a robust systemic inflammatory response, leading to the release of numerous inflammatory mediators (e.g., TNF-α, IL-6) (34). Concurrently, oxidative stress levels rise markedly, inducing cellular and tissue damage and impairing hepatic albumin synthesis (35). These interrelated mechanisms contribute to increased BUN and decreased ALB, ultimately influencing STEMI prognosis.

Limited studies have investigated the effects of BUN and ALB levels at various time points during hospitalization on disease prognosis. A study by Khoury et al. involving 4,768 heart failure patients, found a significantly lower 90-day mortality rate in those with higher BUN at admission that normalized upon discharge compared to those with elevated BUN at both admission and discharge (16.4% vs. 28.8%, *p* < 0.001) (36). In addition, early serum ALB supplementation may reduce the in-hospital mortality associated with sepsis (37). Studies are lacking on whether lowering abnormally elevated BAR levels to the normal range during hospitalization improves in-hospital mortality risk and post-discharge prognosis in NSTEMI patients. Therefore, we propose two key directions for future research: first, investigating whether lowering abnormally high BAR levels improves NSTEMI prognosis; and second, exploring strategies to normalize BAR during hospitalization, particularly by reducing elevated BUN levels, as this may enhance NSTEMI outcomes.

Our study highlights two key points. First, this is the first study investigating the relationship between BAR and in-hospital mortality in Chinese NSTEMI patients, providing novel evidence supporting the global use of BAR as an independent risk factor for in-hospital mortality in NSTEMI. Second, this study is the first to identify a nonlinear relationship between BAR and in-hospital mortality risk in non-diabetic NSTEMI patients, offering a foundation for individualized risk prediction and treatment strategy development.

This study also has several limitations. Firstly, as a secondary analysis of a retrospective study, this research may be affected by various biases and unmeasured confounders. Secondly, certain parameters of the GRACE score, including ST-segment changes on ECG and a history of cardiac arrest, were unavailable. Third, BAR levels may fluctuate during hospitalization. Our study only examined the predictive value of BAR levels at admission for in-hospital mortality in non-diabetic NSTEMI patients, whereas BAR values at different time points or peak BAR values may offer greater prognostic insight. Finally, given that our study is a single-center retrospective study, future large-scale, multicenter prospective cohort studies are required to validate our findings.

## 5 Conclusion

In this study, we found that baseline BAR at admission is a simple and clinically valuable prognostic biomarker for in-hospital mortality in non-diabetic NSTEMI patients. Additionally, we observed a nonlinear relationship with saturation effect between BAR and in-hospital mortality in non-diabetic NSTEMI patients, which may facilitate rapid risk stratification and inform individualized treatment. Further studies are needed to determine whether normalizing abnormally elevated BAR will improve in-hospital outcomes in patients with NSTEMI.

## Data Availability

Publicly available datasets were analyzed in this study. This data can be found here: https://peerj.com/articles/14346.
